# Populational pan-ethnic screening panel enabled by deep whole genome sequencing

**DOI:** 10.1038/s41525-023-00383-8

**Published:** 2023-11-20

**Authors:** Linfeng Yang, Zhe Lin, Yong Gao, Jianguo Zhang, Huanhuan Peng, Yaqing Li, Jingang Che, Lijian Zhao, Jilin Zhang

**Affiliations:** 1 Hebei Industrial Technology Research Institute of Genomics in Maternal and Child Health, BGI-Shijiazhuang Medical Laboratory, Shijiazhuang, China; 2 BGI Genomics, BGI-Shenzhen, Shenzhen, China; 3 Medical Technology College of Hebei Medical University, Shijiazhuang, China; 4 Tung Biomedical Sciences Centre, City University of Hong Kong, Hong Kong S.A.R, China; 5 Department of Precision Diagnostic and Therapeutic Technology, The City University of Hong Kong Shenzhen Futian Research Institute, Shenzhen, China; 6 Department of Biomedical Sciences, City University of Hong Kong, Hong Kong S.A.R, China

**Keywords:** Genomics, Data mining

## Abstract

Birth defect is a global threat to the public health systems. Mitigating neonatal anomalies is hampered by elusive molecular mechanisms of pathogenic mutations and poor subsequent translation into preventative measures. Applying appropriate strategies in China to promote reproductive health is particularly challenging, as the Chinese population compromises complex genomic diversity due to the inclusion of many ethnic groups with distinct genetic backgrounds. To investigate and evaluate the feasibility of implementing a pan-ethnic screening strategy, and guide future reproductive counselling, high-quality variants associated with autosome recessive (AR) diseases derived from the largest publicly available cohort of the Chinese population were re-analysed using a bottom-up approach. The analyses of gene carrier rates (GCRs) across distinct ethnic groups revealed that substantial heterogeneity existed potentially due to diverse evolutionary selection. The sampling population, sequencing coverage and underlying population structure contributed to the differential variants observed between ChinaMAP and the East Asian group in gnomAD. Beyond characteristics of GCR, potential druggable targets were additionally explored according to genomic features and functional roles of investigated genes, demonstrating that phase separation could be a therapeutic target for autosomal recessive diseases. A further examination of estimated GCR across ethnic groups indicated that most genes shared by at least two populations could be utilised to direct the design of a pan-ethnic screening application once sequencing and interpreting costs become negligible. To this end, a list of autosomal recessive disease genes is proposed based on the prioritised rank of GCR to formulate a tiered screening strategy.

## Introduction

The survival rate of children remains a global concern. Although the under-five mortality rate decreased to 3.8% in 2021 from 10% in 1990, a total of five million children under five years old yet have died in 2021, mainly due to congenital anomalies (https://www.who.int/news-room/fact-sheets/detail/levels-and-trends-in-child-under-5-mortality-in-2020). The congenital anomaly, covering a diverse group of disorders that single gene mutations can cause, large structural variations on chromosomes, and other environmental factors, is one of the leading causes of lethality apart from prematurity and infectious diseases^1–3^. Except for a few genetic abnormalities, such as Down’s Syndrome and cystic fibrosis, the causative mechanisms of many birth defects remain poorly understood, including congenital heart defects, cleft palate and club foot. Many causative variants have been characterised and catalogued, further reinforced by large sequencing projects, including the 1000 genome project^4^, the UK Biobank cohort^5^ and the Taiwan Biobank project^6^. Despite the enormous translational potential of identified variants, converting disease-associated variants with intrinsic populational diversity into preventative applications remains challenging, and it still undergoes a primitive exploring phase, especially in preventing congenital anomalies caused by genetic mutations.

Regardless of poorly elucidated underlying pathogenic mechanisms, numerous severe genetic anomalies can be prevented by imposing global or regional surveillance systems and installing preventative carrier screening programs^7–9^. As an effective preventive strategy, the capacity of widely adopted extended carrier screening (ECS) panels has been drastically advanced by the fast-evolving, high-throughput next-generation sequencing techniques, enabling increased access to genetic risk assessment. Indeed, most available screening panels can now focus on 100–200 genetic diseases^10,11^, except for a few larger panels claiming to examine disease-causing variants in 500–600 genes. In addition, because genomic compositions across distinct populations exhibit substantial diversity, carrier frequencies of Mendelian disorders display population-specific characteristics^12^. Thus, many countries or regions have adopted tailored screening panels based on disease prevalence to promote reproductive health, including Mackenzie’s Mission^8^ and Victorian program^13^ in Australia and hemoglobinopathies project in China^9^.

However, one major shortcoming of these ECS schemes is that their screening capacity is restricted because of including a limited number of severe genetic disorders caused by protein-coding variants at a high national-wide or ethnic-specific prevalence^10,11^. The remaining variants and structural variations associated with diseases at a lower prevalence and uncommon/rare diseases are barely considered during the reproductive consultation. Unavoidably, the capacity and scope of these screening panels are yet largely limited and primarily due to the economic consideration regardless of the consideration of population heterogeneity, except for panels to target diseases with high prevalence in the Ashkenazi Jewish population^13^. Moreover, multiple lines of evidence have demonstrated that the gene carrier rate (GCR) of many diseases causing genetic variants shall be considered when implementing the screening strategy. The polyethnic nature of Chinese population calls for a strategical utilisation^14,15^ toward mutation spectrum and ethnic-biased prevalence to deliver integrative reproductive consultation with precision and accuracy.

Nonetheless, the consequences of most observed non-coding variants remain poorly explained. Failing to reveal the underlying molecular mechanisms of their regulatory roles in diseases hinders the search for drug targets, thus the subsequent design of therapeutic strategies. Increasingly reported evidence has proven that non-coding variants play essential roles in pathogenesis due to the dysregulated transcriptional and post-transcriptional processes^16,17^. Since molecular interactions are often compartmentalised, one of the fundamental processes gaining heavy focus, particularly in pathogenesis, is the condensate formation that drives the liquid-liquid phase separation (LLPS)^16,17^. Whether autosomal recessive (AR) disease genes associated with LLPS could be druggable targets is still poorly investigated.

Due to the increased throughput and the drastically decreased cost of sequencing, the turning point of utilising deep whole genome sequencing (WGS) data for personalised and precision reproductive counselling is right on edge. It is now feasible and desirable to use the whole genome data of individuals to expand our capacity to explore the variants from large cohorts to provide a better risk assessment of genetic conditions during reproductive counselling. Indeed, the ECS for prenatal intervention or newborn screening to prevent neonatal disorders have been introduced in many countries^18^, and new tools or approaches to estimate gene carrier rate based on large datasets are emerging^19^. In this study, we leverage the power of ChinaMAP generated through a natural cohort with deep WGS data to demonstrate the benefit of WGS. By comparing GCR estimated from distinct populations, we report the heterogeneous spectra of variants associated with various diseases at a low prevalence in China. More interestingly, analyses of the characteristics of AR disease-causing variants further reveal feasible implementations of tiered pan-ethnic panels considering regional prevalence or rare disease subsets for precision reproduction counselling.

## Results

### Causative variants of autosomal recessive diseases

**Fig. 1:**
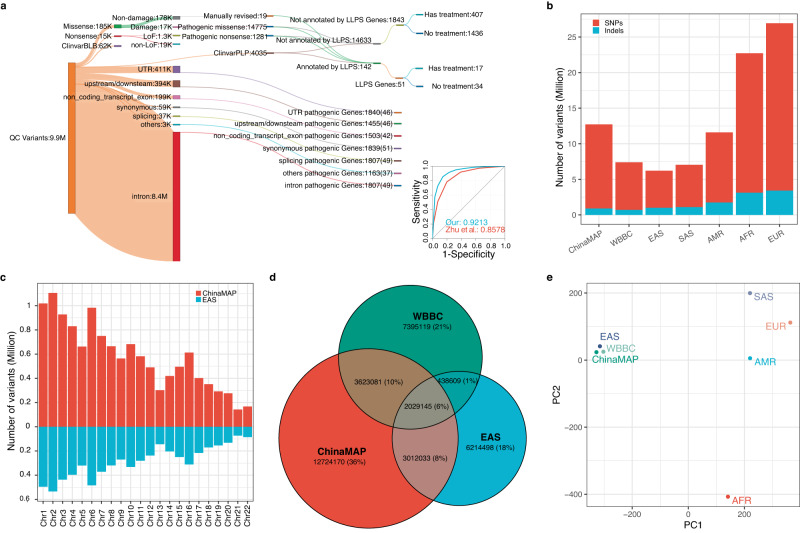
Distribution of variants and identification of AR disease-causing variants. **a** A schematic flow of classifying variants extracted from ChinaMAP. Variants that passed the initial QC are subjected to downstream annotation depending on the nature of genomic loci (protein-coding, intron, splicing junctions, UTR, etc.). The annotated pathogenic variants in protein-coding regions are further divided into the following sub-categories: treatable, untreatable, either associated with LLPS or not, where the number of corresponding genes is shown. To compare the prediction accuracy of missense variants with Zhu et al.’s method, all P/LP and B/LB missense variants extracted from ClinVar (v20221113) are taken as true sets to generate AUC-ROC plot by the R package pROC taking the number of predict tools as threshold. **b** Comparisons of the count of passed-QC variants in distinct ethnic groups included in ChinaMAP and gnomAD databases. **c** The detailed comparison of variant numbers across chromosomes between gnomAD and ChinaMAP. **d** Shared SNPs of East Asian populations between ChinaMAP, gnomAD and WBBC. **e** Principal component analysis based on allele frequency of all studied populations.

To investigate the characteristics of GCR in the Chinese population, we re-analysed variants included in ChinaMAP covering the deep WGS of 10,588 individuals^14^. After quality control, we extracted a total of 140,109,159 variants. To uncover the signature of GCR, 2904 phenotypes corresponding to 2464 AR genes exhibiting Mendelian inheritance were extracted from Online Mendelian Inheritance in Man (OMIM) as candidate genes, where genes associated with syndromes caused by large segmental duplications or chromosomal variations were discarded. The 12.7 million candidate gene overlapping variants were extracted, including 11,826,063 SNPs and 898,110 indels. These variants were subjected to a carefully designed workflow to classify variants as deleterious (Fig. 1a and Supplementary Fig. 1, see “Methods”). We then estimated GCR based on these deleterious variants. Meanwhile, the disease-associating variants were categorised into groups depending on whether treatments were available for the associated conditions/diseases.

We identified 19,484 deleterious variants associated with AR diseases. Among these, 3409 protein-coding variants were annotated by ClinVar database. Except for 19 manually curated variants, 14,775 and 1281 were predicted by our workflow as deleterious missense and deleterious nonsense, respectively (Fig. 1a). Compared to the previously estimated GCR^19^, the GCR reported in our study exhibited a moderate correlation, potentially caused by the intrinsic difference of variants selection using different strategies, cutoffs and prediction tools (Supplementary Figs. 2 and 3). After a systematic comparison, we confirmed that our strategy performed better in predicting P/LP variant as indicated by AUC- ROC (Fig. 1a), and that predicting tools and corresponding thresholds used in this study were strongly evidence-based and widely used, leading to a theoretically lower false discovery rate and a less biased variant set (Supplementary Table 1 and Supplementary Fig. 4). Indeed, GCRs reported here were more consistent with previous designs (Supplementary Fig. 5)^20^. Interestingly, 142 coding variants within 51 AR disease genes were annotated to be involved in LLPS. Among these variants, 65 (53.72%) were catalogued in ClinVar, 55 (45.45%) were deleterious missense variants, and only one was LoF. In contrast, 8.4 million non-coding variants, accounting for 92% of the non-coding variants, were enriched in the intronic regions of 1807 AR disease-causing genes. Unexpectedly, 49 genes were predicted to participate in LLPS. This suggested a promising yet not well-studied direction to disclose the affected regulatory processes beyond misfolded proteins.

As protein-coding sequences only comprised a small portion of the entire genome and many proteins are not targetable by small molecules, we retrieved non-coding variants associated with clear disease-causative genes to explore their potential functional characteristics. Surprisingly, 5.01% and 4.80% of these non-coding variants were enriched within the untranslated regions and up-/downstream of 1891 well-characterised disease-causative genes, respectively. Approximately 36,655 non-coding variants, accounting for 0.45% of the genes associated non-coding variants, were particularly enriched at splicing junctions. Around 45% of these variants were canonical splicing sites, and hundreds of sites were validated by the expression profiles in GTEx (102) (Supplementary Fig. 6) and supported by ICGC (802). However, only 1908, accounting for 5.2% variants, were predicted as splicing-altering sites by SpliceAI (438 acceptor gain, 315 acceptor loss, 269 donor gain, 886 donor loss)^21^. More interestingly, non-coding variants were more enriched in disease-causative genes than non-OMIM genes (Wilcoxon test, *p* < 2.2e-16). These observations implied that post-transcriptional regulation was deeply involved in the pathogenesis apart from that caused by altered protein products.

WBBC and gnomAD were used to assess the power of ChinaMAP data. Indeed, ChinaMAP detected more than 12 million single nucleotide polymorphisms (SNPs), which was over two-fold on average of that for each chromosome in the East Asian ethnic group (EAS) reported by gnomAD (Fig. 1b, c), thus enabling the generation of a much broader and more comprehensive view of the Chinese population. A further comparison of the mutation spectra between ChinaMAP, WBBC and EAS demonstrated that only 43% of gnomAD EAS and 42% of WBBC variants were shared with ChinaMAP, respectively, indicating potentially distinct genetic compositions among datasets (Fig. 1d). Further analysis of allele frequency confirmed that such minor discrepancy did not affect the separation of distant populations (Fig. 1e).

### Estimation of gene carrier rate enhanced by deep WGS in East Asian population

**Fig. 2:**
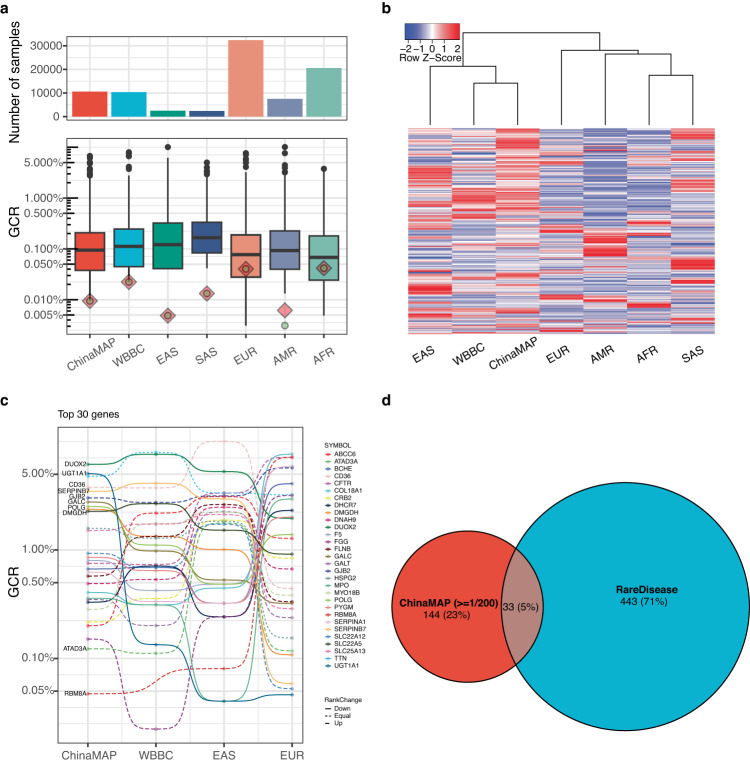
Unique characteristics of GCR estimated from ChinaMAP. **a** Distribution of GCR estimated from all investigated public datasets. Bottom: The distribution of GCR estimated from clear AR disease-causing variants; The green dot and red square represent the GCR that covers 99% and 95% of studied genes, respectively. Top: The corresponding sample size of each ethnic group in the studied databases. **b** Hierarchical clustering of genes with GCR  ≥  1/500 in ChinaMAP are selected to compare with corresponding GCRs in other studied populations. **c** Top 30 genes with drastic GCR changes, the order of which is determined based on the maximum pairwise distance of GCR between populations for a given gene, are selected to demonstrate ethnic-specific GCR features (solid: higher GCR in the East Asian group; dashed-line: lower GCR in the East Asian group). Additionally, the relative rank change of selected genes compared to ChinaMAP is indicated by line type. **d** The overlap of gene lists between ChinaMAP-derived AR disease-causing genes and genes associated with rare diseases officially listed in China.

ChinaMAP covered many diverse minority groups in China, presenting the most comprehensive variations of the East Asian population to date. A substantial number of variants were not detected in the gnomAD EAS population due to technical limits or smaller sampling sizes (Figs. 1b and 2a). Thus, exploiting the dataset to obtain a systematic comparison of GCR was carried out to demonstrate the necessity of WGS to gain improved insight into reproduction counselling. Despite the slightly distinct GCR across populations, many genes of which GCR ≥ 1/500 in different ethnic groups were shared by at least two populations (Fig. 2b and Supplementary Fig. 7), suggesting an ancestral origin that potentially underwent selections^22^. However, the GCR of *DMGDH*, *CD36* and *GJB2*, the variants on which were linked to diseases Dimethylglycine dehydrogenase deficiency, Platelet glycoprotein IV deficiency, and Deafness (autosomal recessive 1A), respectively, exhibited substantial variations across ethnic groups. For instance, variants on gene *CD36* that could cause Platelet glycoprotein IV deficiency were ranked at the top in East Asian groups. In contrast, its observed GCR was drastically lower in several other groups, confirming that diversity caused by potential population-specific selections existed between some groups (Fig. 2c). However, the rank of many genes remained unchanged in other ethnic groups compared to ChinaMAP. In addition, numerous genes with highly ranked GCR were also observed at the leading ends within several other populations, confirming that AR disease genes could commonly affect many ethnic groups other than the non-negligible diversity (Fig. 2b and Supplementary Fig. 7b).

By comparing GCRs estimated from these datasets, we found that the sequencing depth (30X for ChinaMAP, 18X for gnomAD v3) and sample size affected the estimation of GCR in several manners (Fig. 2a). The sample size dictated the detection power, while the under-detected variants caused by insufficient sequencing depth could be compensated by increasing the sampling size^23^. We speculated that the partial congruence with previously estimated GCR of EAS in gnomAD was due to the insufficient detection of variants and potentially false claim of ethnicity. To prove this, we performed PCA analysis on the ChinaMAP, EAS of gnomAD and WBBC at allele frequency level (Fig. 1e) and GCR correlation test between distinct ethnic groups (Supplementary Fig. 8 and Supplementary Table 11) to estimate the impact of low-sequencing coverage and insufficient recovery of rare variants. Indeed, approximately 3.6 million SNPs detected in both ChinaMAP and WBBC were missing in gnomAD, a much larger number compared to that between gnomAD and these two datasets, implying that a lower detection rate of rare variants in the EAS group of gnomAD cohort (Fig. 1d and Supplementary Fig. 9).

After intersecting the selected genes with a list of rare diseases compiled by the Chinese Center for Disease Control and Prevention, we found very few overlapping genes, suggesting that many of the rare diseases lacked appropriate genetic dissections. Alternatively, this potentially indicated the inflation of GCR due to either the carrier effect or heterogeneous genetic composition that could impact the estimation of GCR (Fig. 2c). ChinaMAP uniquely enabled the recovery of genes with higher GCR (Supplementary Fig. 10), confirming that EAS in gnomAD had a distinct genetic background compared to ChinaMAP. We also observed that the estimated GCR of treatable and untreatable diseases across several ethnic groups surprisingly differed significantly (Supplementary Fig. 11). This reinforced the necessity of introducing reproductive counselling for preventative purposes, allocating resources to find novel drug targets, and carrying out innovative research for rare diseases.

### Discovery of potential druggable targets for rare diseases

Although responsible genetic variants of a few neonatal conditions were characterised, many conditions lacked approved treatments, including effective drugs and clinical therapies (Fig. 1a). Most rare diseases are not treatable, calling for unavoidable attention to explore potential druggable targets for these untreatable diseases besides implementing preventative measures.

In addition, the prevalence of certain diseases was primarily dictated by population-specific genetic compositions. Most affected genes varied across the population. For example, genes *UGT1A1*, *SERPINB7* and *ABCG5* were responsible for Crigler-Najjar syndrome, palmoplantar keratosis, phytosterolaemia, exhibited significantly lower GCR in non-Asian ethics compared to that of the Asian ethnic group, suggesting heterogeneous GCR at the population level needed to consider a population-centric priority for the variable genes when implementing preventative strategies or diagnostic strategies^6,12,24^. For investigation, both Asian groups seemed to be more susceptible to diseases Platelet glycoprotein IV deficiency, Lissencephaly 5, Deafness, autosomal recessive 111, Dyssegmental dysplasia, Silverman-Handmaker type/Schwartz-Jampel syndrome, type 1 and Citrullinemia, adult-onset type II / Citrullinemia, type II, neonatal-onset which were caused by mutations in *CD36*, *LAMB1*, *MPZL2*, *HSPG2* and *SLC25A13* supported by a slightly higher GCR compared to other ethnic groups^12,15^, restating the importance of strategic and peculiar drug development to target affected population (Supplementary Fig. 12).

Growing evidence indicated that LLPS participated in various regulatory processes by forming membrane-less organelles in cells, including transcriptional and translational dysregulation in pathogenesis^16,17,25^. As diseases caused by mutations within the protein-coding genes only account for a minor portion of the polymorphism, and many roles of non-coding variants were not examined thoroughly due to their poorly investigated functional roles, we expanded the search scope to identify non-coding variants associated with well-annotated pathogenic genes and potentially dysregulated condensate alterations during LLPS. The rationale was that intrinsically disordered regions pervasively existed in protein sequences and played essential roles in various biological processes through regulating non-membrane organelles. In total, 265,009 variants, accounting for 3.23% of the functionally uncharacterised variants, were involved in LLPS by querying against the list of predicted LLPS associating variants^16^, implying the direction of future drug design and exploration. Indeed, mutations in myosin VIIA and filaggrin could impair their ability to form condensates, leading to subsequent dysregulation of forming motor protein clusters in stereocilia^25^ and assembling keratohyalin granule in keratinocytes, respectively^26^. However, the GCR distribution was not significantly different across populations (Supplementary Fig. 13). A closer examination of the genes that could be impacted by dysregulated LLPS further revealed that at least 60% of diseases without available treatments were associated with the non-membrane organelle forming biomolecules predicted by previously reported approaches^16^ (Supplementary Fig. 14a). The functional enrichment of genes involved in LLPS indicated that genes were associated with extracellular matrix related functions. By performing GO enrichment of 14,487 non-coding variants adjacent to protein-coding genes (UTRs and splicing regions, see “Methods”), we also found that non-coding variants most likely affected genes (83 genes in the top 10 GO) that were involved in amino acid metabolic processes in addition to sensory perception, indicating a potential direction, such as secretome, to search for drug targets. Additionally, we observed that 265 genes fell into the non-treatable category (Supplementary Fig. 14b).

### Rescale pan-ethnic preventative intervention

**Fig. 3:**
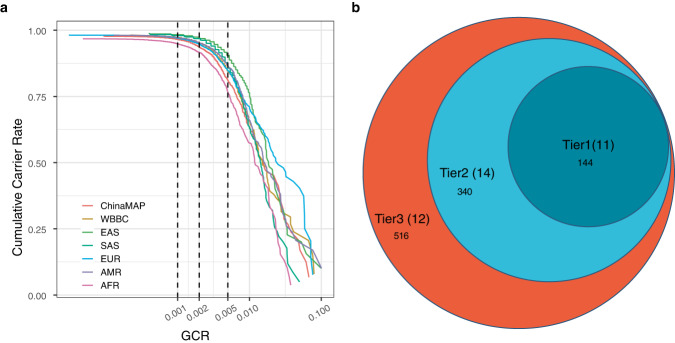
Design of pan-ethnic screening panel. **a** The cumulative carrier rate (CCR) of the top 2000 AR disease-causing genes is visualised. The thresholds of GCR used to select genes are indicated by grey dashed lines. **b** The proposed screening panel with three tiers, the number of included genes (LLPS associated) are chosen based on suggested cutoff 1/200 and 1/500 GCR, and the top 1000.

Finally, we leveraged the power of the WGS-based mutation profile to attempt to exploit its potential in guiding the implementation of preventative measures. The cumulative GCR across distinct populations was surveyed, and it was clear that the number of highly ranked AR-diseases-causing genes were restrained within a certain range regardless of the population-specific genomic composition (Fig. 3a), suggesting that these genes were essential and were potentially selected though they have undergone diverse selection. Based on such observation, genes with a GCR threshold over 1/200 or 1/500 were examined to impute the panel design strategy considering the theoretical prevalence of a disease.

We found that only 20 AR disease-causing genes were exclusively detected by ChinaMAP with a threshold GCR > 1/200 (Supplementary Fig. 7), and all genes prioritised by ChinaMAP were unexpectedly shared with other ethnic groups when the threshold was lowered to GCR > 1/500, potentially because of the aggregated allele frequency of multiple ethnic groups. The comparison of GCRs derived from differential genes and their rankings (Supplementary Fig. 12 and Supplementary Fig. 10) revealed that such difference was likely due to the sampling or intrinsically distinct population structures in the studied populations. These genes showed significantly higher GCR than those in European (EUR) and African (AFR) populations.

Next, a strategy with a three-tier design was proposed to optimise the practical feasibility and rationale (Fig. 3b). Tier 1 and Tier 2 included 144 and 340 genes showing 1/200 and 1/500 GCR in the Chinese population, respectively. However, when the top 1000 genes of each population were extracted for comparison, over 83.26% of AR disease genes were shared by at least two populations (Supplementary Fig. 7c), restating the population heterogeneity could be circumvented once sequencing cost became a minor factor to be considered. The genes in Tier 2 have included genes to be screened during the carrier screening. Indeed, 55% to 80% of genes formed by previously reported panel designs were covered by the proposed panel at Tier 2 (Supplementary Fig. 15 and Supplementary Table 8). However, AR diseases caused by copy number variations, such as in *HBA1*/*HBA2*, *DMD*, *SMN1*, *F8*, *F9*, *IDS*, *MTM1*, *GLA*, *IL2RG* and *OTC* etc., were excluded in our current analysis. Such genes could be selectively supplemented to augment proposed panels based on prevalence in the population.

For genes included in Tier 3, although the theoretical prevalence of their associated AR diseases was much lower in the current Chinese cohort, they could be considered as a complementary list for couples to consider during reproductive counselling and expand the capacity carrier screening panel to a pan-ethnic panel once sequencing cost became neglectable and more reliable medical interpretation could be accessed through advanced or specialised artificial intelligence.

## Discussion

ChinaMAP is one of the largest publicly accessible cohorts providing relatively comprehensive deep WGS-based variant spectra of the Chinese population to understand the heterogeneity, which could potentially guide the implementation of strategies to prevent neonatal anomalies and assist reproductive counselling.

The approach developed in this study aims to assess the feasibility of introducing a pan-ethnic screening strategy focusing on the Chinese population. A thorough comparison with existing approaches has yet to be carried out. However, the correlation between gnomAD EAS and ChinaMAP is at the same level as previously reported observations^19^. An in-depth comparison between approaches demonstrates that GCRs of most genes estimated by ClinVar P/LP showed a high correlation between studies except for a few genes with variants that received major revision of allele frequency in ClinVar. The deviation of GCR could be introduced by several factors, including distinct input data, database revision, variant selection strategy, and heterogeneous strategies applied to predict missense and loss-of function variants. The primary source of discordant GCR estimation in ChinaMAP originates from the variant prediction procedure, where a more stringent strategy in this study has been employed by selecting 100% concordant prediction of nine tools instead, leading to the exclusion of ambiguous variants (Supplementary Fig. 3). Appropriately prioritising genes associated with rare diseases at low prevalence needs to integrate epidemiological information, which is the major limitation once sequencing cost drops to a negligible level.

The substantial difference in estimated GCR between gnomAD and ChinaMAP could be caused by the insufficient sampling bias of the East Asian group included by gnomAD or the heterogeneous compositions of individuals surveyed in ChinaMAP. The sample size of ChinaMAP dataset is four-fold higher than the EAS in gnomAD, which generates the deep WGS data of more than 10 thousand Chinese individuals, granting a more comprehensive capture of the variant spectra. Additionally, the sequencing approach is another potential source of incongruence between datasets as many variants are non-coding variants in ChinaMAP. The higher sequencing coverage used by the ChinaMAP project has a theoretically better capability to detect uncommon variants. It is well-known that China has a complex population structure that involves 55 minority groups. Thus, intrinsic sampling bias could be another source. However, the difference could not be resolved in our study as access to the allele frequency of ChinaMAP individuals was limited. To gain a holistic picture with better socioeconomic benefit, the future screening based on WGS with at 30X shall be considered once the sequencing cost is not a determinant.

The heterogeneous population in China raises concerns on data reusability and translational potential. At the 1/500 GCR threshold, the heterogeneity counterintuitively has refined the power of the screening panel, potentially due to aggregated allele frequency has eliminated population-specific bias caused by selection. Although two-thirds of the genes associated with LLPS have been detected for potential druggable targets, accounting for only a tiny proportion of untreatable diseases, these proteins could be included in the future direction of drug development. One functional study has demonstrated that mutations in *MYO7A* weakens its ability to form *MYO7A*/*USH1C*/*USH1G* complex which impairs phase separation, resulting in an abnormal tip-link densities and causing hearing and vision loss in Usher syndrome patients^25^. Another example is that mutations in filaggrin (*FLG*), which are associated with human skin barrier disorders, could alter the properties of proteins leading to an abolished formation of keratophyalin granules that can subsequently compromise skin defence^26^, although the role of mutated *FLG* in AR is yet to be characterised. Nevertheless, many exciting discoveries related to LLPS remain to be clarified with substantial research on the mutation affected LLPS and require clear dissection of corresponding mechanistic insights. More innovative approaches will be invented to target these variants to find solutions for rare diseases^27^. The resulting knowledge from our analysis could relieve the socioeconomic burdens and, more importantly, benefit families carrying untreatable rare diseases. The detailed molecular mechanism, however, requires more experiments to disclose the altered functional roles in cells, which is beyond the scope of this study.

Finally, interpreting the present screening results of ECS to assist reproduction counselling in China depends on prior knowledge established based on non-Chinese populations. This potentially could cause biased evaluations and lead to undesired reproductive decisions. Our analyses provide a more compressive view of the GCR in the Chinese population to guide the design of preventative measures and even drug searching direction. As the advance in sequencing techniques will continuously reduce the sequencing cost, biobanks with larger cohorts and more comprehensive information will emerge rapidly and globally. The rationale and feasibility of implementing a pan-ethnic screening strategy for preventative reproductions also need to consider the rapid iteration of artificial intelligence. With an automated, precise and yet reliable interpretation driven by state-of-art artificial intelligence algorithms, the economic cost will eventually reduce to a level where almost all rare diseases can be screened to promote human reproductive health.

## Methods

### Candidate genes of autosomal recessive diseases

To focus on the autosomal recessive (AR) disorder caused by SNPs/Indels, a list of candidate genes was collected from OMIM database (https://www.omim.org/downloads) based on the following rules:

- includes AR-associated genes,
- excludes genes labelled by non-disease phenotypes or associated with multifactorial disorders, or the relationship between phenotype and gene is provisional,
- exclude genes of which phenotype is a chromosome deletion or duplication syndrome.

In total, 2464 candidate AR genes were obtained to perform subsequent analysis (Supplementary Table 1).

### Variants collection of different population

The China Metabolic Analytics Project (ChinaMAP) dataset was used to estimate the GCR of AR disorders in the Chinese population. The ChinaMAP project has released a variants frequency spectrum of 10,588 Chinese individuals determined by deep whole genome sequencing^14^, a site-only VCF containing allele frequencies was downloaded from mBiobank (www.mbiobank.com/download/). To obtain high-quality variants, QUAL ≤ 100 was discarded, common variants with AF ≥ 0.05 were also filtered out. In addition, to narrow down the margin error of low allele frequency (0.001) to ± 0.0005 in 95% of interval confidence, allele with AN < 10,229 was filtered out^28^.

The gnomAD^29^ v3.1.2 data was used to compare the difference of GCR among populations with distinct genomic compositions. Allele frequencies of candidate genes of different subgroups stored in the VCF files were collected from gnomAD (https://gnomad.broadinstitute.org/downloads). The ‘Non-Cancer’ group was used to calculate GCR. Variants without “PASS” tag in the FILTER column or AF > = 0.05 were discarded. A list of candidate variants of five populations, including EAS, SAS, EUR, AMR and AFR, was selected by setting at least one allele cutoff (AC > 0) for each population.

The Westlake BioBank for Chinese (WBBC) cohort^30^, which recruited 14,726 participants in the pilot project, was included to survey the potential diversity within the Chinese population. The samples were primarily collected from Jiangxi, Shandong and Zhejiang provinces. Site-only VCFs of autosome chromosomes were also obtained (https://wbbc.westlake.edu.cn/downloads.html). All variants in the files were considered high quality.

### Variants annotation

All variant files from public databases were normalised using bcftools (bcftools norm -m -)^31^ before being annotated by VEP^32^ with ensemble database release version 108, the most severe transcript of each gene was considered to identify potential deleteriousness. The ClinVar^33^ database (release version V20221113) was also applied to annotate the variants. An in-house script was used to extract variants located in the candidate genes. These subsets of annotated variants were used to identify the subsequent deleterious variants.

### Deleterious variants

Deleterious variants of each population were identified separately following a carefully designed workflow (Supplementary Fig. 1). First, variants annotated as benign or likely benign by ClinVar were discarded, while those annotated as pathogenic or likely pathogenic with at least one star of review status were kept. The remaining variants, including pathogenic/likely pathogenic with conflicting annotation in ClinVar database, were further classified into three subgroups: (i). missense variants were predicted by nine computational tools with general cutoff: CADD ≥ 20^34,35^, Eigen≥1^36^, REVEL ≥ 0.75^37^, DANN > = 0.5^38,39^, Polyphen2 = =’D’^40^, SIFT = = ’D’^41^, MetaSVM = =’D’^42^, MutationAssessor = =‘H’ or ‘M’^43,44^, PROVEAN == ‘D’. Only variants with all tools passed the cutoff were considered as deleterious. (ii). nonsense variants (including frameshift_variant, stop_gained, splice_donor_variant, splice_acceptor_variant and start_lost), which classified as highly-confident loss-of-function by LOFTEE^29^, were predicted by autoPVS1^45^ in advance. Only variants with ‘Strong’ or ‘Very Strong’ adjusted strength were considered as deleterious. (iii). non-missense/nonsense variants were discarded. Tools and corresponding cutoff settings listed above were carefully chosen based on the performance reported by previous studies^46,47^ (Supplementary Table 10). Moreover, we filtered out variants by gene based on AF. For genes with at least one pathogenic variant identified by ClinVar, the max AF of ClinVar variant was used as a cutoff, otherwise using 0.005. Finally, for the ChinaMAP dataset, we manually revised missense variants that failed by the above process but with conflicting pathogenic/likely pathogenic interpretations in the ClinVar database. All putatively deleterious variants of each population were listed in Supplementary Tables 2–8.

### Candidate autosomal recessive gene annotation

To distinguish untreatable diseases from treatable diseases, we searched for potential treatments from “Treatments for genetic disorders” (www.rx-genes.com) by the name of genes carrying deleterious variants. Genes with any treatment returned by the database were considered as treatable, otherwise untreatable. Additionally, we used a set of deleterious variants, including predicted (missense and LoF) variants and those catalogued in ClinVar, that affect liquid-liquid phase separation (LLPS) as described in Salman F Banani’s study^16^, to identify genes related to non-membrane organelle formation. If a deleterious variant in this study was also reported by Banani et al., the corresponding gene was considered as LLPS gene.

### GO enrichment

To investigate the potential of drug development, a set of LLPS-related genes which contain more than 200 adjacent non-coding variants (including 5′- and 3′-UTR, up- and downstream and splicing region variants) were collected for GO enrichment using clusterProfiler^48^, all significantly enriched (*p*-value < 0.01 and adjust *p*-value < 0.05) GO terms were ranked according to the count of input genes for visualisation.

### Gene carrier rate

To estimate GCR, the variant carrier rate (VCR) was first calculated according to Eq. (1) introduced in^49^:

1 $${VCR}=1-\frac{{AC}-2* {Hom}}{0.5* {AN}}$$

*Hom* represents the number of homozygous individuals. To facilitate the calculation, a simplified estimation was applied to all candidate genes under the assumption that homozygous variants are rare for severe AR conditions, where *Hom* count could be ignored. To confirm this hypothesis, GCRs with and without accounting for homozygous of the WBBC dataset were used to investigate the effect of *Hom* alleles. Assuming that homozygous individuals only accounted for a relatively small proportion of a population, the results showed that the two calculation methods are almost identical (Supplementary Fig. 16). Hence, the GCR can be estimated through Eq. (2):

2 $${{GCR}}_{g}=1-\mathop{\prod }\limits_{i=0}^{v}\left(1-{VC}{R}_{{\rm{i}}}\right)=1-\mathop{\prod }\limits_{i=0}^{v}(1-\frac{{AC}}{0.5* {AN}})$$

GCR of each population was summarised in Supplementary Tables 2–8.

### Panel design and comparison

To assess the robustness of proposed screening panels selected based on distinct cutoffs, two potential panels described by Xi et al.^50^ and Wei et al.^6^ were used to carry out a thorough comparison (Supplementary Tables 2–8, Supplementary Fig. 15).

### Reporting summary

Further information on research design is available in the Nature Research Reporting Summary linked to this article.

### Supplementary information

Supplementary materials

Supplementary Tables 1-11

REPORTING SUMMARY

## Data Availability

The datasets used and/or analysed during the current study are included in this published article as supplementary data files or reproducible through the provided source code.

## Code availability

The code for this study is available on GitHub and can be accessed via the link: https://github.com/LinZhe-SZ/CHN_ECS.

## References

[1] Dolk H, Loane M, Garne E (2010). The prevalence of congenital anomalies in Europe. Adv. Exp. Med. Biol..

[2] Ostrander B, Bale JF (2019). Congenital and perinatal infections. Handb. Clin. Neurol..

[3] Chen LJ, Chiou JY, Huang JY, Su PH, Chen JY (2020). Birth defects in Taiwan: a 10-year nationwide population-based, cohort study. J. Formos. Med. Assoc..

[4] Genomes Project C (2015). A global reference for human genetic variation. Nature.

[5] Halldorsson BV (2022). The sequences of 150,119 genomes in the UK Biobank. Nature.

[6] Wei CY (2021). Genetic profiles of 103,106 individuals in the Taiwan Biobank provide insights into the health and history of Han Chinese. NPJ Genom. Med..

[7] Gregg AR (2021). Screening for autosomal recessive and X-linked conditions during pregnancy and preconception: a practice resource of the American College of Medical Genetics and Genomics (ACMG). Genet. Med..

[8] Kirk EP (2021). Gene selection for the Australian Reproductive Genetic Carrier Screening Project (“Mackenzie’s Mission”). Eur. J. Hum. Genet..

[9] Zhao S (2019). Pilot study of expanded carrier screening for 11 recessive diseases in China: results from 10,476 ethnically diverse couples. Eur. J. Hum. Genet..

[10] Arjunan A (2020). Evaluation and classification of severity for 176 genes on an expanded carrier screening panel. Prenat. Diagn..

[11] Beauchamp KA (2018). Systematic design and comparison of expanded carrier screening panels. Genet. Med..

[12] Xiao Q, Lauschke VM (2021). The prevalence, genetic complexity and population-specific founder effects of human autosomal recessive disorders. NPJ Genom. Med..

[13] Leibowitz R (2022). Reproductive genetic carrier screening for cystic fibrosis, fragile X syndrome and spinal muscular atrophy: patterns of community and healthcare provider participation in a Victorian screening program. Aust. J. Prim. Health.

[14] Cao Y (2020). The ChinaMAP analytics of deep whole genome sequences in 10,588 individuals. Cell Res..

[15] Pan Y (2023). Comparative genomic and transcriptomic analyses reveal the impacts of genetic admixture in Kazaks, Uyghurs, and Huis. Mol. Biol. Evol..

[16] Banani SF (2022). Genetic variation associated with condensate dysregulation in disease. Dev. Cell.

[17] Mensah MA (2023). Aberrant phase separation and nucleolar dysfunction in rare genetic diseases. Nature.

[18] Seydel C (2022). Baby’s first genome. Nat. Biotechnol..

[19] Zhu W (2022). A robust pipeline for ranking carrier frequencies of autosomal recessive and X-linked Mendelian disorders. NPJ Genom. Med..

[20] Johansen Taber K (2022). A guidelines-consistent carrier screening panel that supports equity across diverse populations. Genet. Med..

[21] Jaganathan K (2019). Predicting splicing from primary sequence with deep learning. Cell.

[22] Karlsson EK, Kwiatkowski DP, Sabeti PC (2014). Natural selection and infectious disease in human populations. Nat. Rev. Genet.

[23] Fumagalli M (2013). Assessing the effect of sequencing depth and sample size in population genetics inferences. PLoS ONE.

[24] Hanks SC (2022). Extent to which array genotyping and imputation with large reference panels approximate deep whole-genome sequencing. Am. J. Hum. Genet..

[25] He Y, Li J, Zhang M (2019). Myosin VII, USH1C, and ANKS4B or USH1G Together Form Condensed Molecular Assembly via Liquid-Liquid Phase Separation. Cell Rep..

[26] Quiroz FG (2020). Liquid-liquid phase separation drives skin barrier formation. Science.

[27] Greene D (2023). Genetic association analysis of 77,539 genomes reveals rare disease etiologies. Nat. Med.

[28] Cochran, W. G. *Sampling Techniques* 3rd edn.

[29] Karczewski KJ (2020). The mutational constraint spectrum quantified from variation in 141,456 humans. Nature.

[30] Cong PK (2022). Genomic analyses of 10,376 individuals in the Westlake BioBank for Chinese (WBBC) pilot project. Nat. Commun..

[31] Danecek P (2021). Twelve years of SAMtools and BCFtools. Gigascience.

[32] McLaren W (2016). The ensembl variant effect predictor. Genome Biol..

[33] Landrum MJ (2018). ClinVar: improving access to variant interpretations and supporting evidence. Nucleic Acids Res..

[34] Rentzsch P, Schubach M, Shendure J, Kircher M (2021). CADD-Splice-improving genome-wide variant effect prediction using deep learning-derived splice scores. Genome Med..

[35] Niroula A, Vihinen M (2019). How good are pathogenicity predictors in detecting benign variants?. PLoS Comput. Biol..

[36] Ionita-Laza I, McCallum K, Xu B, Buxbaum JD (2016). A spectral approach integrating functional genomic annotations for coding and noncoding variants. Nat. Genet..

[37] Ioannidis NM (2016). REVEL: an ensemble method for predicting the pathogenicity of rare missense variants. Am. J. Hum. Genet..

[38] Quang D, Chen Y, Xie X (2015). DANN: a deep learning approach for annotating the pathogenicity of genetic variants. Bioinformatics.

[39] Zeng Z, Aptekmann AA, Bromberg Y (2021). Decoding the effects of synonymous variants. Nucleic Acids Res..

[40] Adzhubei I, Jordan DM, Sunyaev SR (2013). Predicting functional effect of human missense mutations using PolyPhen-2. Curr. Protoc. Hum. Genet..

[41] Ng PC, Henikoff S (2003). SIFT: predicting amino acid changes that affect protein function. Nucleic Acids Res..

[42] Kim S, Jhong JH, Lee J, Koo JY (2017). Meta-analytic support vector machine for integrating multiple omics data. BioData Min..

[43] Reva B, Antipin Y, Sander C (2011). Predicting the functional impact of protein mutations: application to cancer genomics. Nucleic Acids Res..

[44] Choi Y, Chan AP (2015). PROVEAN web server: a tool to predict the functional effect of amino acid substitutions and indels. Bioinformatics.

[45] Xiang J, Peng J, Baxter S, Peng Z (2020). AutoPVS1: an automatic classification tool for PVS1 interpretation of null variants. Hum. Mutat..

[46] Katsonis P, Wilhelm K, Williams A, Lichtarge O (2022). Genome interpretation using in silico predictors of variant impact. Hum. Genet..

[47] Li J (2018). Performance evaluation of pathogenicity-computation methods for missense variants. Nucleic Acids Res..

[48] Wu T (2021). clusterProfiler 4.0: a universal enrichment tool for interpreting omics data. Innovation.

[49] Guo MH, Gregg AR (2019). Estimating yields of prenatal carrier screening and implications for design of expanded carrier screening panels. Genet. Med..

[50] Xi Y (2020). Expanded carrier screening in Chinese patients seeking the help of assisted reproductive technology. Mol. Genet. Genom. Med..

