# Supplementary material for: Populational pan-ethnic screening panel enabled by deep whole genome sequencing
Source: NPJ Genom Med. 2023 Nov 20;8:38. doi: 10.1038/s41525-023-00383-8 (PMC10661700; doi:10.1038/s41525-023-00383-8)
Supplement: Supplementary file 1 — Supplementary materials [file 41525_2023_383_MOESM1_ESM.pdf]

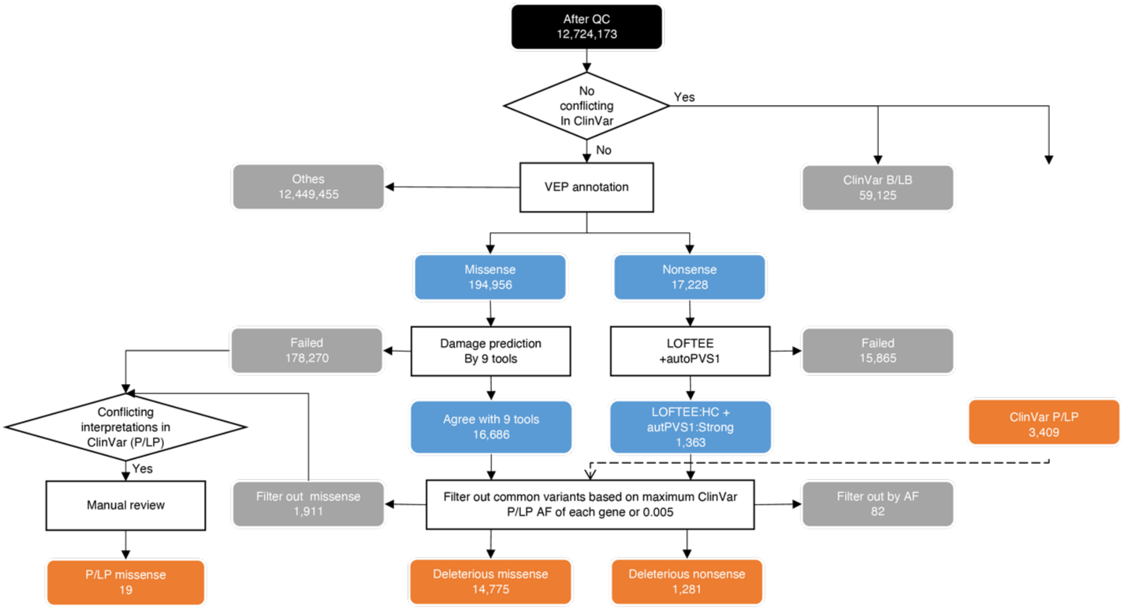

Supplementary Figure 1. Pathogenic mutation identification workflow.
A schematic workflow of identifying pathogenic mutation from the ChinaMAP dataset. First, QC is performed on the released site-only VCF, and around 12 million variants pass the criteria (See Methods). Next, based on ClinVar annotation, 3,409 variants are identified as 'Pathogenic/Likely Pathogenic' while 59,125 were 'Benign/Likely Benign'. Then by taking into account the most severe transcript, 194,956 missense and 17,228 nonsense variants are obtained. Missense variants that are predicted concordantly as damage by nine tools and nonsense with confident evidence by LOFTEE and autoPVS1 are retained. These missense/nonsense variants are filtered by allele frequency (AF) using the max AF of ClinVar P/LP of the corresponding gene or 0.005 as a cutoff. Finally, 14,775 damage missense and 1,281 pathogenic nonsense variants have been identified. Moreover, those missense variants with 'Conflicting' P/LP by ClinVar annotation failing to pass the filter are manually revised, and 19 variants are retrieved. Other populations in this study are processed similarly, except for the QC criteria (**See Methods**).

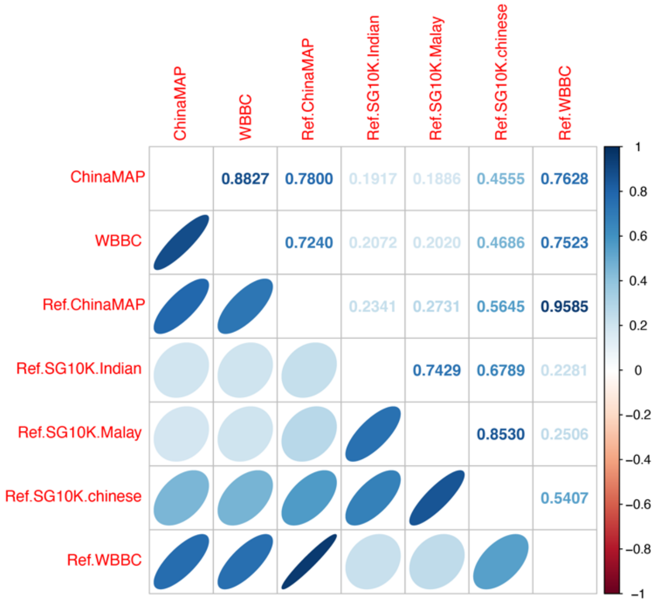

Supplementary Figure 2. The comparison of estimated GCR by our proposed approach and previously reported method [1].

The previously reported GCR was named with the prefix "Ref". The upper and lower matrices of the back-diagonal both present the Pearson correlation coefficients but with different visual representations (numbers and ellipse charts).

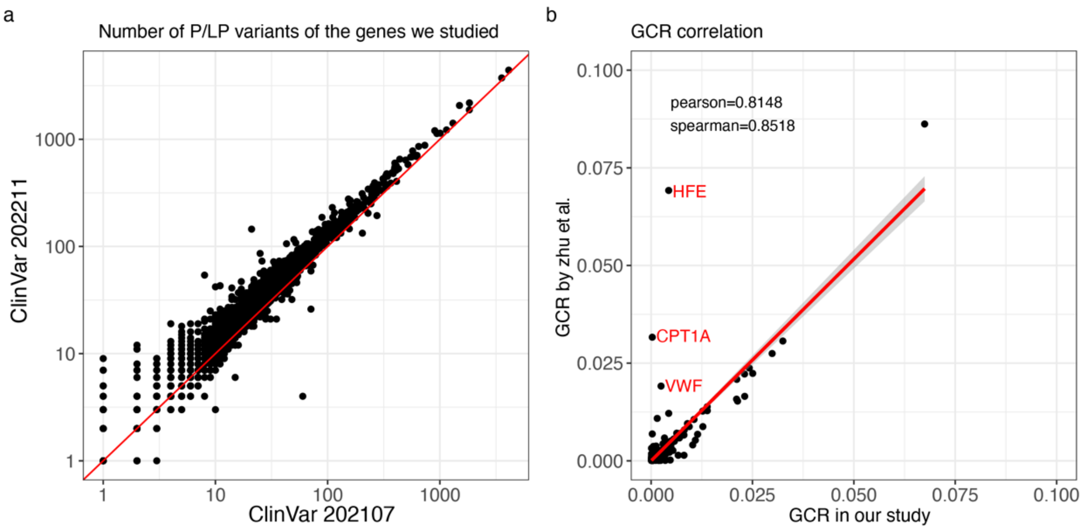

Supplementary Figure 3. Variant number and GCR comparison between ClinVar versions.

a: The number of P/LP variants of two ClinVar versions used by Zhu et al. and ours. b: GCR correlation between two studies when considering ClinVar variants only.

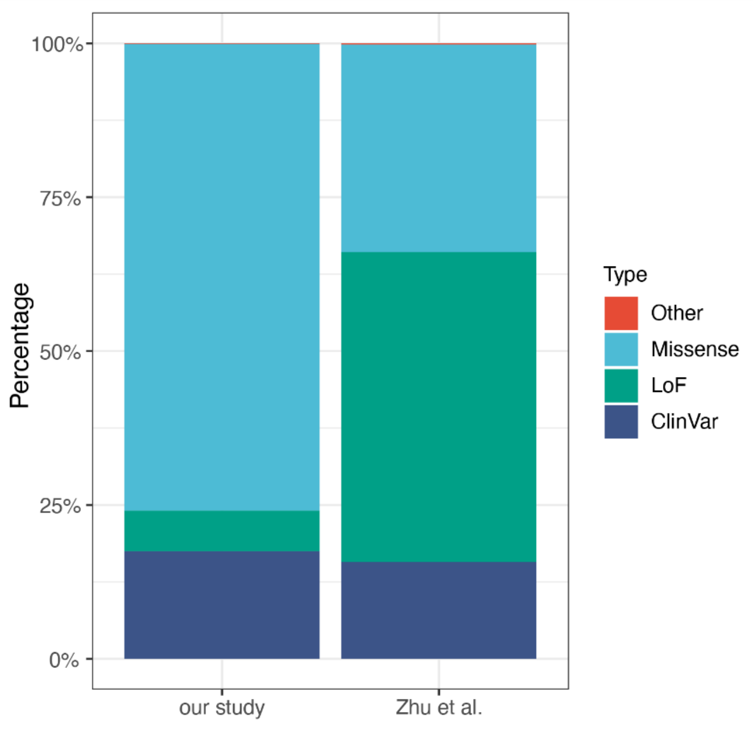

Supplementary Figure 4. Percentage of deleterious variants reported by Zhu et al. and our study.

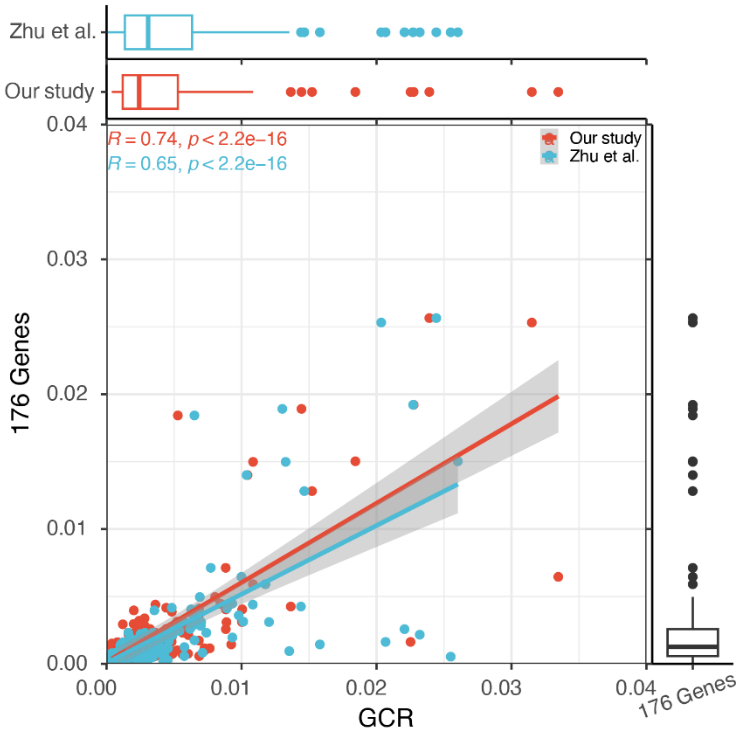

Supplementary Figure 5. **GCR correlation for 176 previously reported genes between studies**.

The GCRs of 176 genes reportedly previously are compared with the GCR estimated by Zhu et al. and the approach described in this study (Pearson correlation).

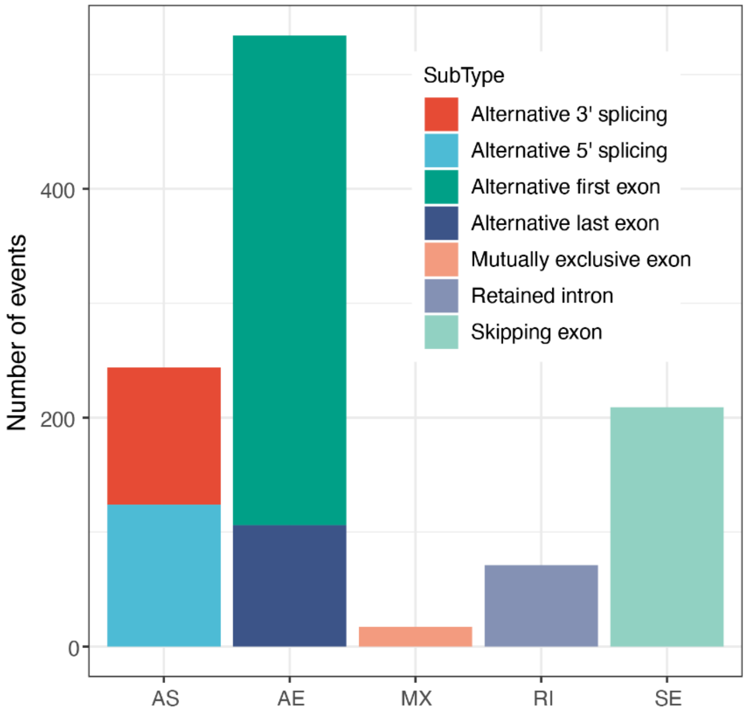

Supplementary Figure 6. Number of splicing events validated by GTEx dataset.
We observed 102 non-coding variants in GTEx's sQTL datasets. SUPPA2 (https://github.com/comprna/SUPPA/) is used to calculate percentages of splicing inclusion (PSI) from 17,382 RNA-seq individuals, and finally defined 1,075 splicing events (not applicable PSIs less than 20 and variance of PSIs greater than 0.001), which is related to those non-coding variants, including seven different sub-types.

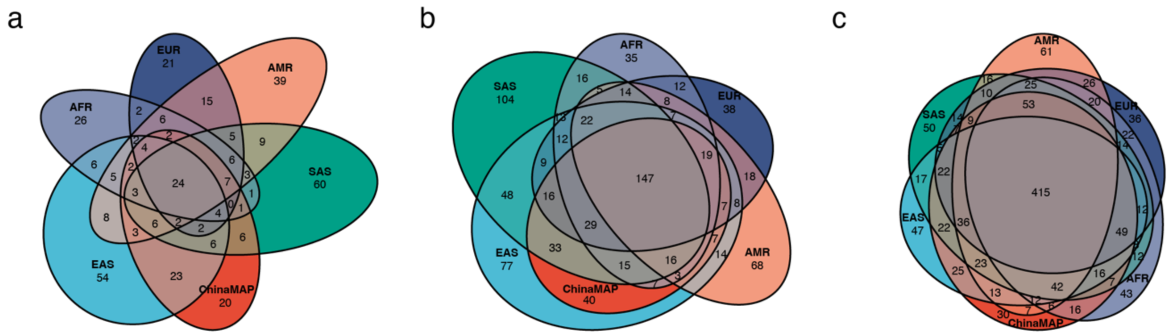

Supplementary Figure 7. Overlap of pathogenic genes above various GCR thresholds for the investigated populations.

The GCR of each gene is calculated after pathogenic variants are identified. The Venn diagrams show the overlap of pathogenic genes among the five studied populations based on the following cutoffs: a. Selected genes with GCR≥0.005, b. Selected genes with GCR≥0.002, c. The top 1000 genes ranked based on GCR in each studied population.

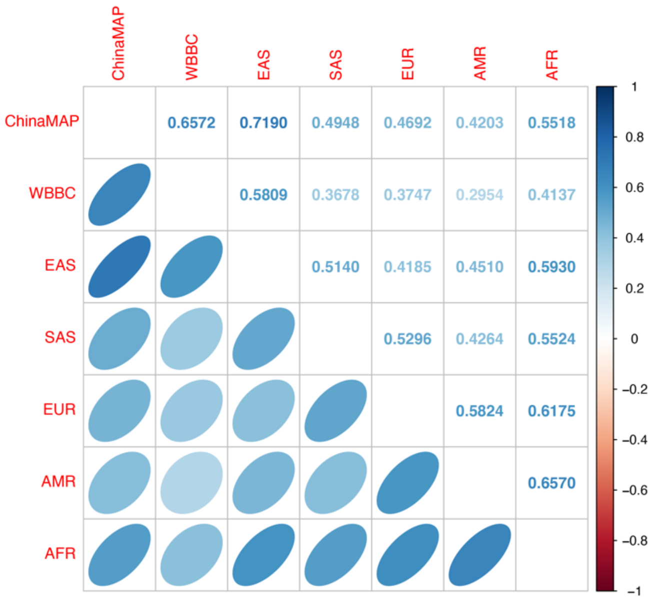

Supplementary Figure 8. **GCR correlation between distinct ethnic groups.**
Pearson correlation of GCR between different ethnic groups. The representation is using the same approach described in Supplementary Figure 2.

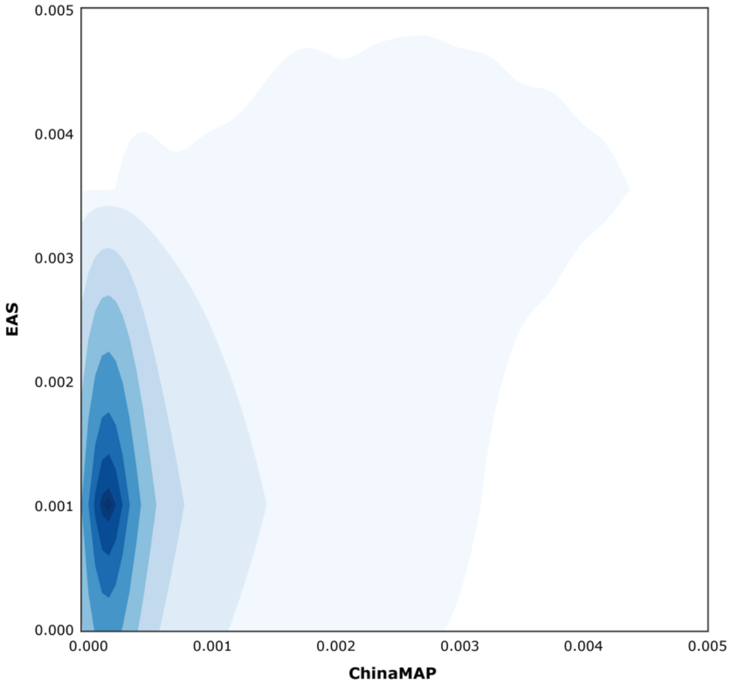

Supplementary Figure 9. Comparison of AF of rare variants between ChinaMAP and EAS of gnomAD.

The distribution of AFs of rare variants (AF<0.005) is roughly consistent between ChinaMAP and EAS of gnomAD, except a small fraction of variants in EAS is observed with biasedly higher AF. This is potentially due to the smaller sample size and lower sequencing depth of EAS.

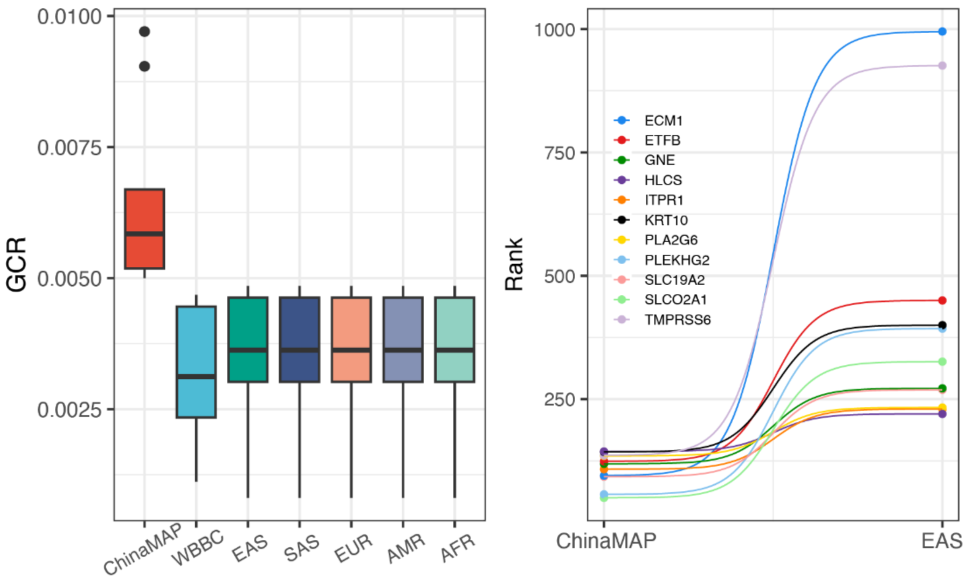

Supplementary Figure 10. Comparison of unique genes with GCR≥1/200 in ChinaMAP. Twenty genes unique to ChinaMAP with GCR≥1/200 show higher GCR than other populations. The rank of these unique genes ranges from 3 to 144, while all are ranked below 220 in EAS.

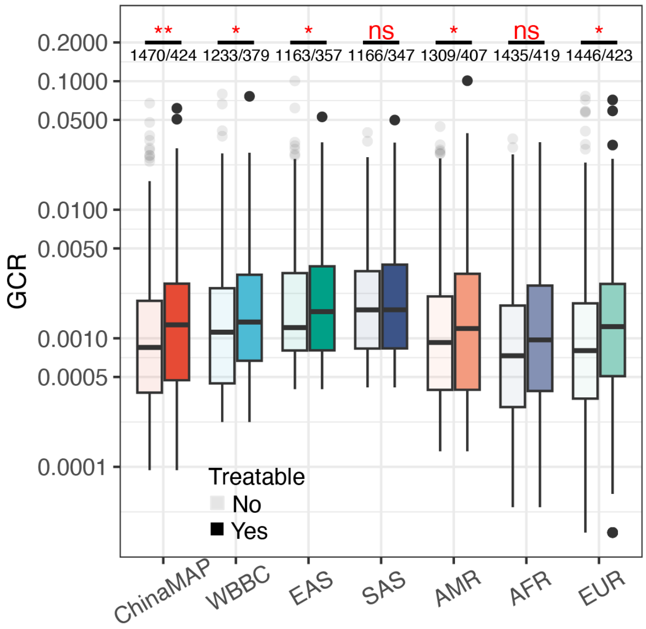

Supplementary Figure 11. GCR distribution of populations group by treatable.

GCR distribution of populations grouped by the availability of treatment. Potential treatments are queried from "Treatments for genetic disorders" (www.rx-genes.com) using gene names to classify genes into treatable and untreatable categories. * and ** denote the significance between treatable and non-treatable with *t-test* at significance levels of 0.05 and 0.01, respectively; ns represents not-significantly different.

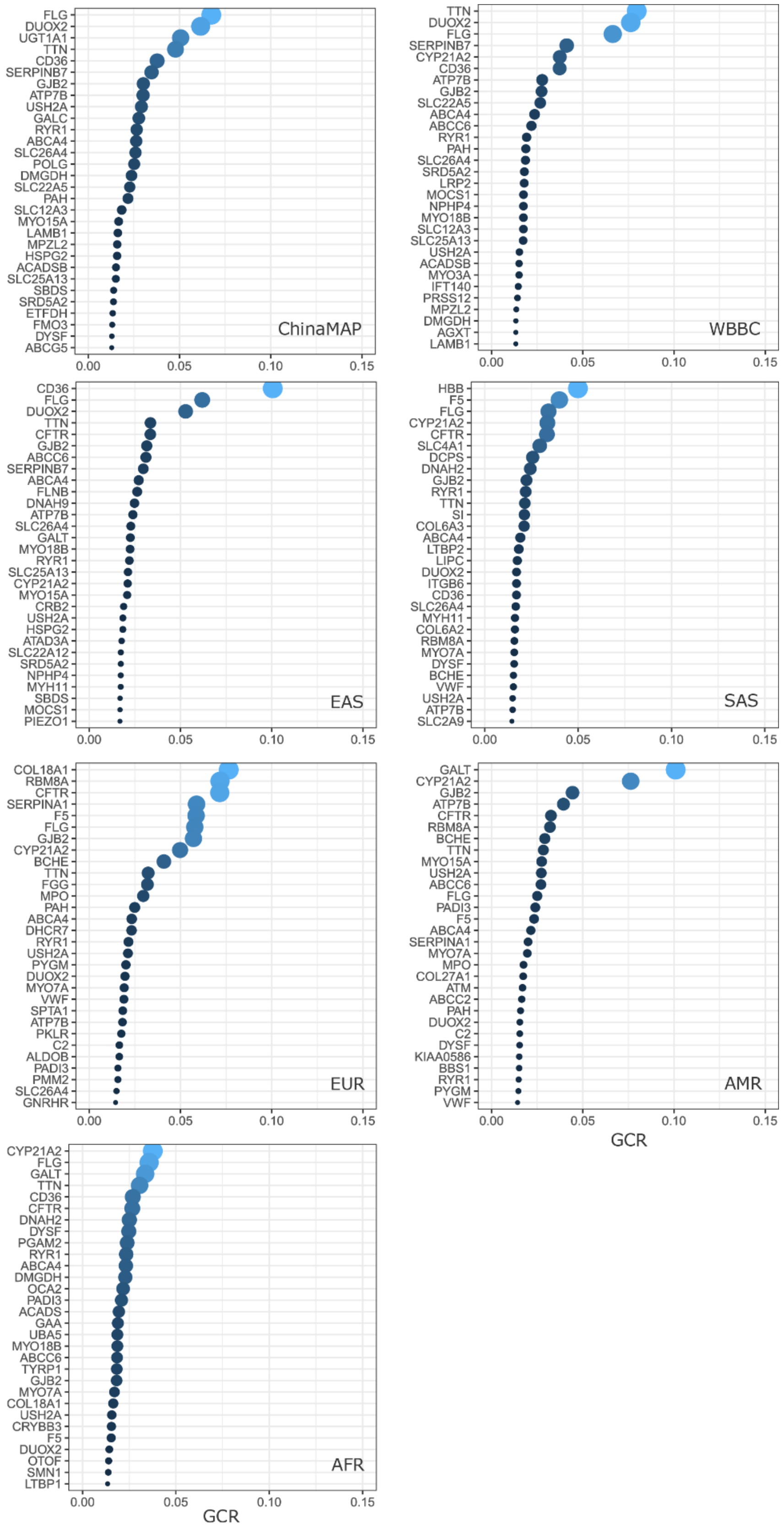

Supplementary Figure 12. Top 50 Genes ordered by GCR in six populations. The top 50 genes ranked according to the GCR of six studied populations.

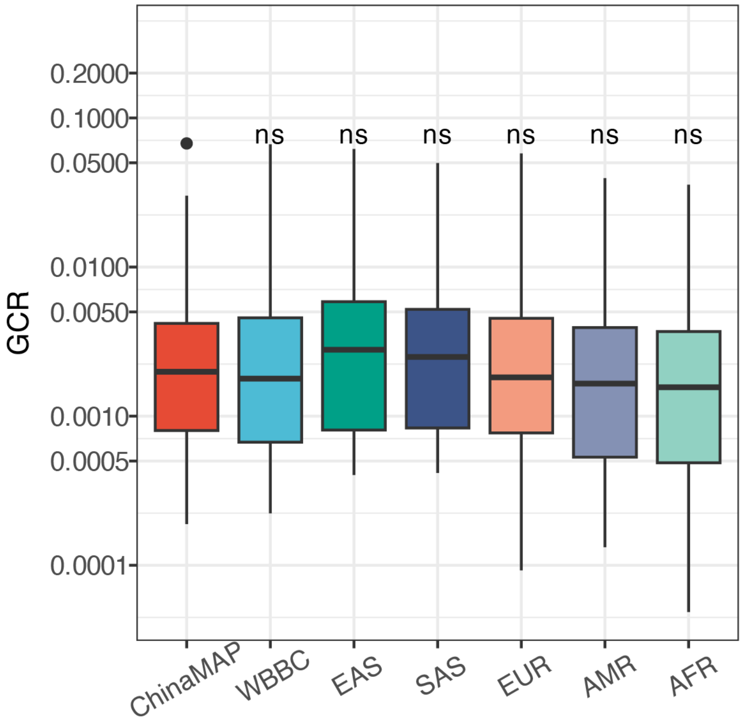

Supplementary Figure 13. GCR of LLPS-associating genes.

Boxplot of GCR of 51 LLPS-associating genes among populations.

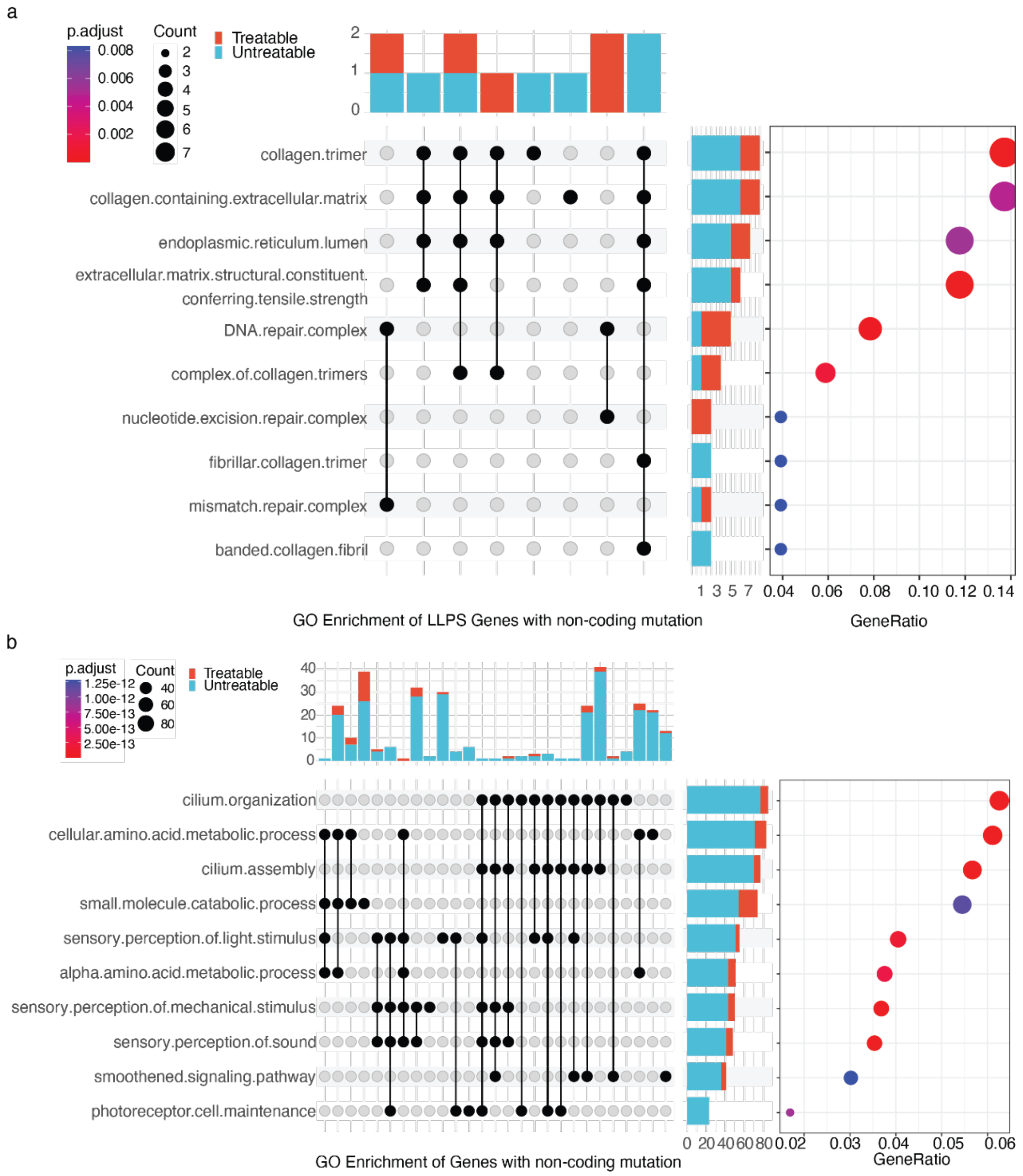

Supplementary Figure 14. GO enrichment genes with or adjacent to non-coding variants. a) GO enrichment of genes with adjacent non-coding variants. b) GO enrichment of LLPS-related genes with non-coding variants. In total, 51 LLPS-related genes with non-coding variants were collected to perform GO enrichment.

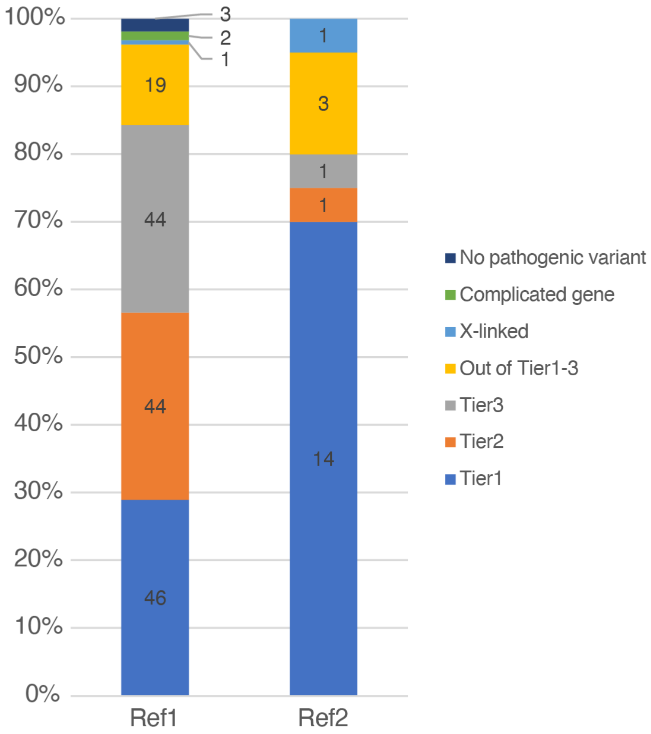

Supplementary Figure 15. Panel design compared with two other publications. Comparison of the proposed panel design strategies with panels described in Xi et al. [2,3] (Ref1) and Wei et al. [2] (Ref2). Genes from all three tiers are considered for comparison. A small fraction of genes (three from Ref1 and one from Ref2) are not considered in our design because they are X-linked or complicated for WGS technology. Notably, three genes of Ref1 are missing in this study because no pathogenic variants have been identified.

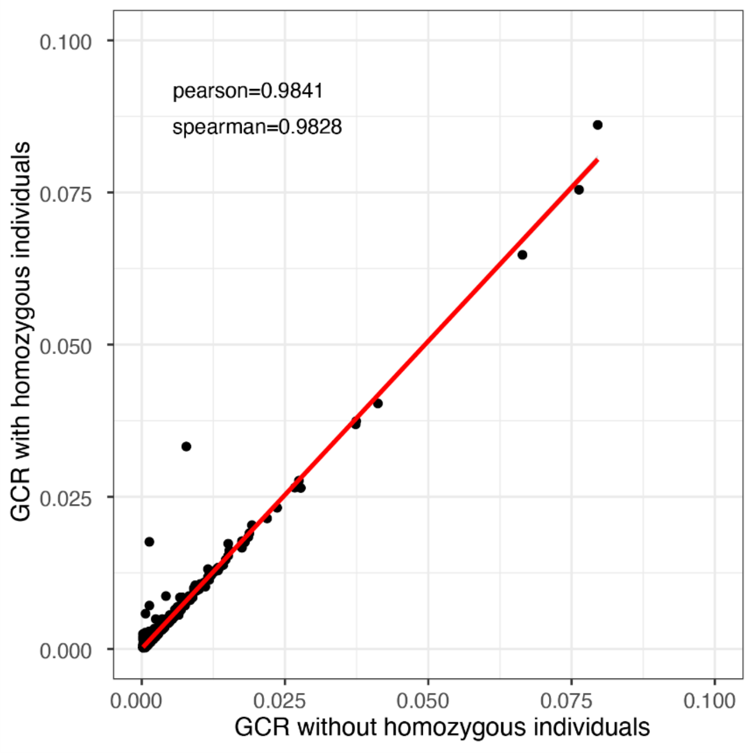

Supplementary Figure 16. Correlation between calculating GCR with/without accounting for homozygous.

Reference

1 Zhu, W., Wang, C., Mullapudi, N., Cao, Y., Li, L., Lo, I. F. M., Tsui, S. K., Chen, X., Lei, Y. & Gu, S. A robust pipeline for ranking carrier frequencies of autosomal recessive and X-linked Mendelian disorders. *NPJ Genom Med* **7**, 72 (2022).

2 Xi, Y., Chen, G., Lei, C., Wu, J., Zhang, S., Xiao, M., Zhang, W., Zhang, Y. & Sun, X. Expanded carrier screening in Chinese patients seeking the help of assisted reproductive technology. *Mol Genet Genomic Med* **8**, e1340 (2020).

3 Wei, C. Y., Yang, J. H., Yeh, E. C., Tsai, M. F., Kao, H. J., Lo, C. Z., Chang, L. P., Lin, W. J., Hsieh, F. J., Belsare, S., Bhaskar, A., Su, M. W., Lee, T. C., Lin, Y. L., Liu, F. T., Shen, C. Y., Li, L. H., Chen, C. H., Wall, J. D., Wu, J. Y. & Kwok, P. Y. Genetic profiles of 103,106 individuals in the Taiwan Biobank provide insights into the health and history of Han Chinese. *NPJ Genom Med* **6**, 10 (2021).

Supplementary Tables

Supplementary Table 1. Candidate genes

Supplementary Table 2-8. Variants of ChinaMAP, populations included in gnomAD and WBBC

Supplementary Table 9. Raw data for panel comparison in Supplementary Figure 9

Supplementary Table 10. List of prediction tools with applied threshold

Supplementary Table 11. Correlation between populations
