# Supplementary material for: Populational pan-ethnic screening panel enabled by deep whole genome sequencing
Source: NPJ Genom Med. 2023 Nov 20;8:38. doi: 10.1038/s41525-023-00383-8 (PMC10661700; doi:10.1038/s41525-023-00383-8)
Supplement: Supplementary file 3 — REPORTING SUMMARY [file 41525_2023_383_MOESM3_ESM.pdf]

nature portfolio

Corresponding author(s): Jilin Zhang

Last updated by author(s): Aug 18, 2023

Reporting Summary

Nature Portfolio wishes to improve the reproducibility of the work that we publish. This form provides structure for consistency and transparency in reporting. For further information on Nature Portfolio policies, see our Editorial Policies and the Editorial Policy Checklist.

Statistics

For all statistical analyses, confirm that the following items are present in the figure legend, table legend, main text, or Methods section.

n/a	Confirmed	
☐	☒	The exact sample size (*n*) for each experimental group/condition, given as a discrete number and unit of measurement
☒	☐	A statement on whether measurements were taken from distinct samples or whether the same sample was measured repeatedly
☐	☒	The statistical test(s) used AND whether they are one- or two-sided<br>*Only common tests should be described solely by name; describe more complex techniques in the Methods section.*
☒	☐	A description of all covariates tested
☐	☒	A description of any assumptions or corrections, such as tests of normality and adjustment for multiple comparisons
☒	☐	A full description of the statistical parameters including central tendency (e.g. means) or other basic estimates (e.g. regression coefficient) AND variation (e.g. standard deviation) or associated estimates of uncertainty (e.g. confidence intervals)
☒	☐	For null hypothesis testing, the test statistic (e.g. *F*, *t*, *r*) with confidence intervals, effect sizes, degrees of freedom and *P* value noted<br>*Give P values as exact values whenever suitable.*
☒	☐	For Bayesian analysis, information on the choice of priors and Markov chain Monte Carlo settings
☒	☐	For hierarchical and complex designs, identification of the appropriate level for tests and full reporting of outcomes
☒	☐	Estimates of effect sizes (e.g. Cohen's *d*, Pearson's *r*), indicating how they were calculated

Our web collection on statistics for biologists contains articles on many of the points above.

Software and code

Policy information about availability of computer code

Data collection	vcftools 1.16 was used to processing VCFs, VEP v108 was used to annotate variants, spliceAI v1.3.1 was used to identify splice variants. SUPPA2 was used to generate splice events and calculate PSI, a custom python script was used to determine deleterious variation and calculate GCR, GO enrichment was performed by R package 'clusterProfiler', all figures were plotted by R package ggplot2 in R 4.1 environment.
Data analysis	analysis scripts in our study has been deposit on github: https://github.com/LinZhe-SZ/CHN_ECS.

For manuscripts utilizing custom algorithms or software that are central to the research but not yet described in published literature, software must be made available to editors and reviewers. We strongly encourage code deposition in a community repository (e.g. GitHub). See the Nature Portfolio guidelines for submitting code & software for further information.

Data

Policy information about availability of data

All manuscripts must include a data availability statement. This statement should provide the following information, where applicable:

- Accession codes, unique identifiers, or web links for publicly available datasets
- A description of any restrictions on data availability
- For clinical datasets or third party data, please ensure that the statement adheres to our policy

All the data in our study is publicly available:

- variants of three population research projects were downloaded from their website: ChinaMap (www.mbiobank.com/download/), gnomAD (https://gnomad.broadinstitute.org/downloads), WBBC (https://wbbc.westlake.edu.cn/downloads.html)

Research involving human participants, their data, or biological material

Policy information about studies with human participants or human data. See also policy information about sex, gender (identity/presentation), and sexual orientation and race, ethnicity and racism.

Reporting on sex and gender	This information has not been collected.
Reporting on race, ethnicity, or other socially relevant groupings	Ethnic population information was defined by each population research project. Our study did not refer to any individual information
Population characteristics	This information has not been collected.
Recruitment	This information is not relevanted in our study.
Ethics oversight	This study do not involve any individual information and were approved by Institutional Review Board of BGI.

Note that full information on the approval of the study protocol must also be provided in the manuscript.

Field-specific reporting

Please select the one below that is the best fit for your research. If you are not sure, read the appropriate sections before making your selection.

☒ Life sciences ☐ Behavioural & social sciences ☐ Ecological, evolutionary & environmental sciences

For a reference copy of the document with all sections, see nature.com/documents/nr-reporting-summary-flat.pdf

Life sciences study design

All studies must disclose on these points even when the disclosure is negative.

Sample size	Our study used variant allele frequency data of public population research projects, including The ChinaMap project which contain 10,588 Chinese individuals determined by deep whole-genome sequencing, gnomAD which contains thousands of different populations, The Westlake BioBank for Chinese (WBBC) cohort, which recruited 14,726 participants in the pilot project
Data exclusions	no data were excluded from the analyses.
Replication	no replicated, we compare our method with previous results to validate the method is effective
Randomization	not relevant to our study because did not designed any experimental group.
Blinding	not relevant to our study.

Reporting for specific materials, systems and methods

We require information from authors about some types of materials, experimental systems and methods used in many studies. Here, indicate whether each material, system or method listed is relevant to your study. If you are not sure if a list item applies to your research, read the appropriate section before selecting a response.

Materials & experimental systems

n/a	Involved in the study	
☒	☐	Antibodies
☒	☐	Eukaryotic cell lines
☒	☐	Palaeontology and archaeology
☒	☐	Animals and other organisms
☒	☐	Clinical data
☒	☐	Dual use research of concern
☒	☐	Plants

Methods

n/a	Involved in the study	
☒	☐	ChIP-seq
☒	☐	Flow cytometry
☒	☐	MRI-based neuroimaging
